# Advances in the effectiveness and safety of azvudine treatment: a comprehensive review

**DOI:** 10.3389/fphar.2025.1524072

**Published:** 2025-04-25

**Authors:** Jiayi Li, Bo Zhu, Jian Lu, Zheyi Dong, Ping Li, Wenge Li, Chunfu Zheng, Junbiao Chang, Shunlai Shang

**Affiliations:** ^1^ Pingyuan Laboratory, Xinxiang, China; ^2^ Department of Nephrology, China-Japan Friendship Hospital, Beijing, China; ^3^ Department of Nephrology, First Medical Center of Chinese PLA General Hospital, Nephrology Institute of the Chinese People’s Liberation Army, National Key Laboratory of Kidney Diseases, National Clinical Research Center for Kidney Diseases, Beijing Key Laboratory of Kidney Disease Research, Beijing, China; ^4^ Department of Microbiology, Immunology and Infectious Diseases, University of Calgary, Calgary, AB, Canada

**Keywords:** azvudine, COVID-19, SARS-CoV-2 RdRp, pharmacokinetics, special patients

## Abstract

The global impact of COVID-19 has highlighted the urgent need for effective therapeutic interventions against SARS-CoV-2. Azvudine, a dual-target nucleoside drug initially developed for human immunodeficiency virus (HIV), has gained attention for its potential in treating COVID-19. On 25 July 2022, Azvudine received conditional approval from the National Medical Products Administration (NMPA) of China, making it the first oral SARS-CoV-2 RNA-dependent RNA polymerase (RdRp) inhibitor for COVID-19 treatment. This review explores the pharmacological activity, antiviral mechanisms, and clinical effectiveness of azvudine in the context of COVID-19. Clinical trials have demonstrated its ability to reduce the viral load, shorten the time to nucleic acid negativity, and improve clinical outcomes in patients. Additionally, azvudine has shown excellent pharmacokinetic properties and a favorable safety profile with mild side effects. The review also addresses the importance of drug interactions and safety considerations, particularly in high-risk populations. Research should focus on optimizing second-generation inhibitors with enhanced effectiveness against SARS-CoV-2 variants, improving oral bioavailability, and minimizing adverse effects, ensuring more robust treatment options for COVID-19.

## 1 Introduction

The emergence of severe acute respiratory syndrome coronavirus 2 (SARS-CoV-2) has driven the rapid development and approval of several antiviral drugs to mitigate the impact of COVID-19. Among these, RNA-dependent RNA polymerase (RdRp) inhibitors and protease inhibitors have played a crucial role in reducing viral replication, disease severity, and mortality. Notable oral antivirals such as Remdesivir, Molnupiravir, and Nirmatrelvir/Ritonavir (Paxlovid), Azvudine have been widely used in clinical practice.

**FIGURE 1:**
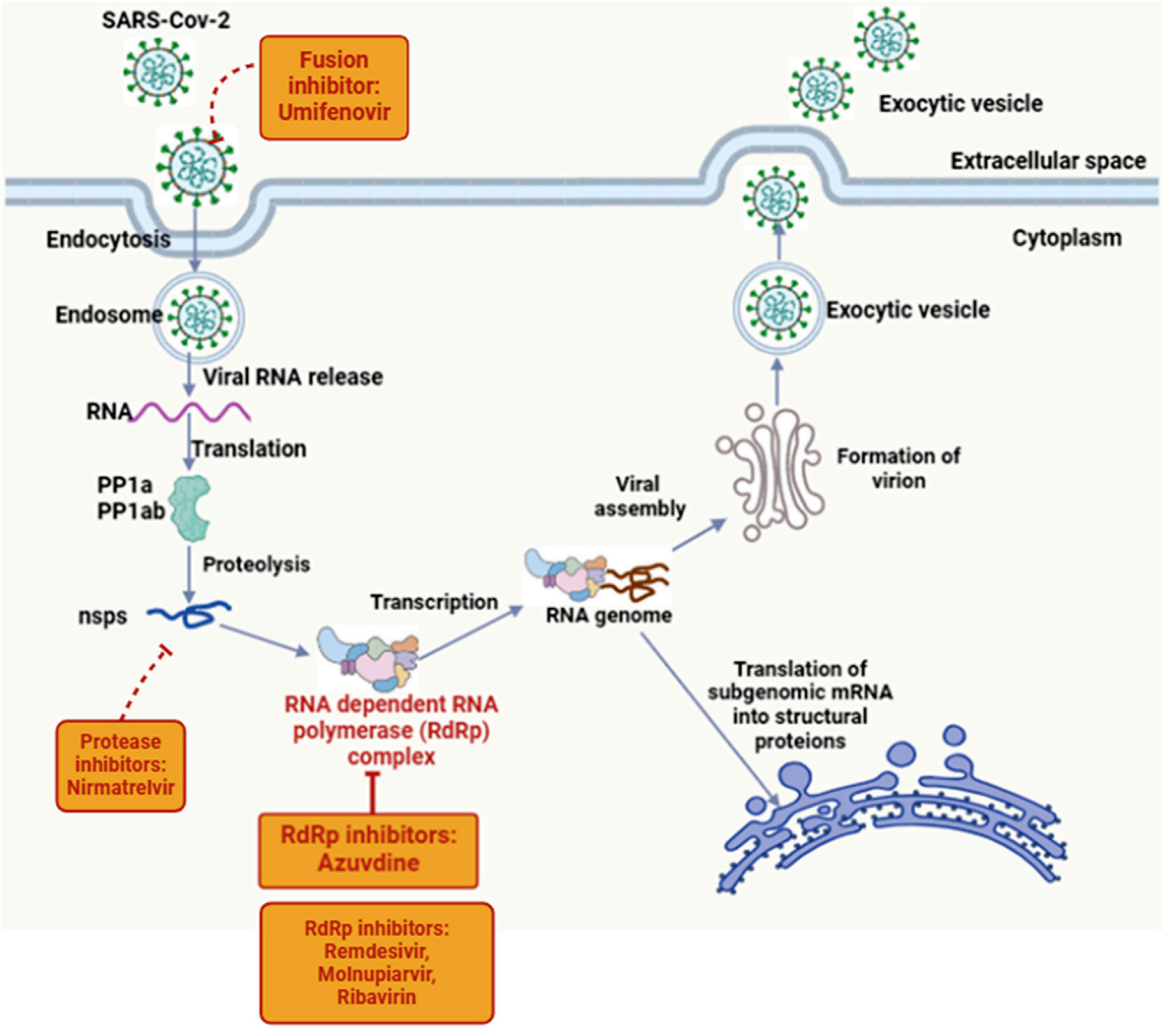
Overview of the life cycle of SARS-CoV-2 and targets for the antiviral drug azvudine. In the host cell, the spike protein (S) of SARS-CoV-2 interacts with the cellular receptor ACE2, followed by viral entry into the host cell. The virus enters the host cell in two ways, either through serine proteases that activate the virus to fuse with the plasma membrane, or through the host’s endocytic machinery that activates the virus to fuse with the cell membrane. The viral genome is released in the cytoplasm and translated into the viral replicase polyproteins (PP1a and PP1ab), which are subsequently cleaved by the viral proteases to form non-structural proteins (nsps). Some of the nsps then form the replicase-transcriptase complex, the RNA-dependent RNA polymerase (RdRp). Through intermittent transcription, the polymerase produces a family of subgenomic mRNAs that are ultimately translated into functional viral proteins. In the cytoplasm, the viral nucleocapsid is formed along with genomic RNA and N proteins, and buds in the lumen of the ERGIC. The virus is then released from the host’s infected cells into the extracellular space by exocytosis. Created with BioRender software (https://app.biorender.com/)

Azvudine is the first dual-target nucleoside drug (inhibiting nucleoside reverse transcriptase and restoring cytidine deaminase expression) with broad-spectrum antiviral activity against human immunodeficiency virus (HIV), hepatitis C virus (HCV), enterovirus 71 (EV71), and hepatitis B virus (HBV) infections (Zhang J. L. et al., 2021). It is also known to modulate P-glycoprotein (P-gp) expression and is effective against SARS-CoV-2 (Liu et al., 2017; Liu et al., 2018). Its chemical name is 1-(4-azido-2-deoxy-2-fluoro-β-D-arabinofuranosyl) cytosine, with a molecular formula of C9H11FN6O4 and a relative molecular weight of 286.22 (Yu and Chang, 2022). RdRP is a promising therapeutic target for SARS-CoV-2 infection. Azulfidine (FNC, RO-0622), a first-in-class nucleoside-based prodrug developed by Henan Sincere Biotech Co., Ltd., received conditional marketing authorization from the National Drug Administration (NMPA) on 25 July 2022, becoming the first oral SARS-CoV-2 RdRp inhibitor for treating adult COVID-19 patients in China (Yu and Chang, 2022; Yu and Chang, 2020). Figure 1 shows the life cycle of SARS-CoV-2 and the targets of azvudine. Recent clinical trials have shown that 40% of people treated with azvudine have improved clinical symptoms (NCT04668235) (Zhang J. L. et al., 2021). Following these results, Chinese authorities approved azvudine for COVID-19 treatment. The drug has demonstrated desirable pharmacokinetic properties, excellent effectiveness, and safety in initial clinical trials (NCT04303598, CXHS2000016, CXHS2000017). During the clinical studies, COVID-19 patients received 5 mg per day of azvudine combined with standard treatment for up to 14 days (NCT05033145, NCT04668235). Real-world evidence has provided critical insights into their performance outside controlled clinical settings. A real-world study analyzed Remdesivir’s effectiveness, confirming significant clinical improvement and viral load reduction (Mazzitelli et al., 2023a). Another observational study reported Molnupiravir and nirmatrelvir/ritonavir’s real-life data on tolerability, safety, and adverse eventsin high-risk populations (Mazzitelli et al., 2023b). Additionally, a multicenter real-world study showed the use of oral antivirals to treat COVID-19 was associated with a reduced risk of hospitalization and inpatient disease progression among older patients living in nursing homes (Ma et al., 2023). A summary of the dose, frequency, clinical indications and effectiveness of antivirals and mAbs in COVID-19 patients is shown in Table 1.

**TABLE 1:** Dosage, clinical indications and effectiveness of antivirals and monoclonal antibodies in COVID-19 patients.

Class/drugs	Dosage/route/duration and clinical indications	Patients characteristics/recommendations	Clinical effectiveness in COVID-19
RNA-dependent RNA polymerase (RdRp) inhibitor			
Remdesivir	Mild-to-moderate COVID-19 patients: 200 mg i.v. On the 1st day, 100 mg for the next 2–3 days	Mild-to-moderate COVID-19 patients	Shortens the time of recovery from COVID-19
	Severe COVID-19: above treatment can be extended up to 10 days	Hospitalized and severe COVID-19 patients	Lowers respiratory tract-related complications
	In adults with eGFR ≥60: 4 mg/kg, orally/day	In COVID-19 patients with eGFR ≥60 mL/min/1.73 m2	
	In pediatric patients, 5 mg/kg on day 1 followed by 2.5 mg/kg		
Molnupiravir	Moderate to severe COVID-19: 800 mg/oral/12 h for 5 days	Moderate to severe COVID-19 patients	Lowers the risk of hospitalization
		COVID-19 patients with mild-to-moderate kidney dysfunction	Decreased the % death among unvaccinated adults
		Patients with influenza and coronavirus infections	
Famciclovir	Dose ranges from 125 to 1500 mg/day (depending on the status of kidney function in COVID-19 patients)	COVID-19-associated pneumonia	Inhibits cell-mediated inflammation and immune cells activations
		COVID-19 patients with kidney impairment or hemodialysis (with low dose)	Accelerates viral clearance and prevents swelling and vesiculation
		Patients with herpes simplex virus infections (varicella zoster and herpes zoster)	Gradually improves kidney functions
Ribavirin	COVID-19: 400 mg/twice/daily when taken with lopinavir/ritonavir or 500 mg twice/thrice/day	Mild-to-moderate COVID-19 patients without kidney impairment	Shortens the duration of hospitalizations
	Hepatitis-C: 800–1200 mg/day	Hepatitis-C patients	Increases the effectiveness of lopinavir/ritonavir
Favipiravir	COVID-19: maintenance dose of 200–600 mg twice daily and various loading doses of 1600, 1800, and 2400 mg for 10 days	COVID-19	No promising results in COVID-19 patients with or without kidney dysfunction
	Influenza: 1600 mg twice daily on day1 and 600 mg twice daily from day2–5	Influenza	
Protease inhibitors			
Atazanavir	COVID-19: 300–400 mg/day/oral along with other antivirals	COVID-19 and COVID-19 with moderate kidney impairment or receiving dialysis	Improves oxygen saturation, clinical and paraclinical characteristics of COVID-19
	HIV infection: 400 mg/oral/day or 300 plus 100 mg of ritonavir/oral/day	HIV.	
Darunavir	COVID-19: 800 mg with 150 mg of cobicistat per day for 5–7 days along with conventional therapies	COVID-19 and COVID-19 with kidney impairment	Controversial outcomes regarding the effectiveness and safety
Nirmatrelvir/ritonavir combination	COVID-19: a) Patients having eGFR ≥60 mL/min: 300 mg of nirmatrelvir and 100 mg of ritonavir twice daily for 5 days	COVID-19 and COVID-19 with kidney impaired patients having eGFR ≥30 mL/min	Significantly lowered the mortality rate. Reduces the risk of hospitalization or progression to severe COVID-19 infection in vaccinated patients
	b) Patients having an eGFR ≥30–60 mL/min: 150 mg og nirmatrelvir and 100 mg of ritonavir twice daily for 5 days		
Lopinavir/ritonavir combination	COVID-19: 400 mg (lopinavir) and 100 mg (ritonavir) per day/oral for 10 days	COVID-19 and COVID-19 with eGFR >90 mL/min/1.73 m^2^ as acute therapy	No confident data is available regarding the clinical effectiveness
		Chronic hepatitis B or C	
Neuraminidase inhibitors			
Oseltamivir	COVID-19: 75 mg once or twice a day for 5–14 days, alone or in combination with drugs like HCQS or azithromycin	COVID-19 and COVID-19 with CKD or ESKD patients wherein TDM is required	Shorten hospital stays and reduced the mortality rate among hospitalized COVID-19 patients
		Influenza A and influenza B viruses	
Nucleoside reverse transcriptase inhibitors (NRTIs)			
Azvudine	COVID-19: 5 mg/oral/day in combination with standard treatment for up to 14 days	COVID-19 and COVID-19 with HIV or CKD patients	Improved COVID-19 related clinical symptoms
		HIV, HCV, EV71, and HBV infections	Reduces viral load, inflammation, and organ damage
HIV integrase inhibitors/non-nucleoside reverse transcriptase inhibitors (NNRTIs)			
Dolutegravir/Rilpivirine	In HIV patients with or without COVID-19: 40–50 mg/day up to 10 days	HIV patients with or without COVID-19	Higher activated partial thromboplastin time, and lower C-reactive protein and potassium level
		COVID-19 with CKD, ESKD or dialysis wherein TDM is required	Suppressed viral load and cause less severe disease course
Nucleotide polymerase inhibitors			
Sofosbuvir	COVID-19: Sofosbuvir 400 mg with ravidasvir 200 mg or daclatasvir 60 mg, orally for 7–10 days	COVID-19.Hepatitis-C	Improved clinical symptoms, oxygen saturation, and decreased incidence of mortality in moderate to severe COVID-19 patients
	Hepatitis-C: 400 mg/day and generally given in combination with ribavirin, and velpatasvir		
Fusion inhibitors			
Umifenovir (Arbidol)	COVID-19: 800 mg twice a day for a maximum of 14 days	COVID-19 (Mild-asymptomatic).Influenza A and B	Mild-asymptomatic COVID-19 patients were found clinically recovered and RT-PCR negative
Monoclonal antibodies			
Bamlanivimab/Etesevimab (BEC)	COVID-19: 700 mg of bamlanivimab and 1400 mg of etesevimab	COVID-19 with or without kidney impairment	Lower rate of hospitalization and reduced need for any supplementary oxygen
Casirivimab/Imdevimab	COVID-19: Casirivimab 2400 mg and imdevimab 1200 mg, single intravenous treatment	COVID-19 with or without CKD and dialysis	Significant reduction in viral load, hospitalizations and all-cause mortality
Sotrovimab and Tocilizumab	COVID-19: Sotrovimab (500 mg) and tocilizumab (8 mg/kg)	COVID-19 and kidney transplant recipient	Reduction in cytokines level, inflammation and all over clinical symptoms
Baricitinib	COVID-19: 2–4 mg/day for 14 days	COVID-19	Decreased immune cells activation and mortality rate
Adalimumab and Sarilumab	COVID-19: Adalimumab- 40 mg with SoC. Sarilumab- single 400 mg, i.v. Or 200 mg in divided dose	COVID-19 with or without CKD and mild-moderate kidney failure	Decreased cytokine synthesis, inflammation and improvement in clinical symptoms and all-cause mortality rate

Abbreviations* COVID-19: Coronavirus Disease-2019, CKD: chronic kidney disease, ESKD: end stage kidney disease, HCQS: hydroxychloroquine, i. v.: Intravenous GFR: glomerular filtration rate, TDM: therapeutic drug monitoring, HIV: human immunodeficiency virus, HCV: hepatitis C virus, EV71: enterovirus 17, HBV: hepatitis B virus, SoC: standard of care, RT-PCR: reverse transcription-polymerase chain reaction test.

Compared to other COVID-19 drugs, azvudine offers several unique advantages. Its dual-target mechanism provides a broader antiviral spectrum and potentially reduces the risk of drug resistance (Wang et al., 2025; Meng et al., 2024). Additionally, its ability to modulate P-glycoprotein expression may enhance its therapeutic effects and safety profile. The molecular mechanism of azvudine involves its incorporation into viral RNA by RdRp, leading to chain termination and inhibition of viral replication. This mechanism is distinct from some other COVID-19 drugs that target different stages of the viral life cycle, such as viral entry or assembly.

## 2 Search strategy

A comprehensive literature search was conducted to identify relevant studies on azvudine’s effectiveness, safety, and mechanism of action in the treatment of COVID-19. The following databases were systematically searched: PubMed, Web of Science, Embase, and Cochrane Library. The search covered articles published up to 2025/3/31, using a combination of keywords and Medical Subject Headings (MeSH) terms, including: (“Azvudine” OR “FNC” OR “RO-0622″) AND (“COVID-19″OR “SARS-CoV-2″), (“Nucleoside analog” OR “RNA-dependent RNA polymerase inhibitor”) AND (“COVID-19 treatment”). The inclusion criteria were: 1. Studies investigating the pharmacokinetics, effectiveness, and safety of azvudine. 2. Clinical trials, real-world studies, and preclinical investigations. 3. Articles published in English. The exclusion criteria were: 1. Non-peer-reviewed articles and preprints without sufficient data validation.2. Studies focusing on unrelated diseases or mechanisms. Additional references were identified through manual screening of bibliographies from relevant publications. Two independent reviewers assessed the eligibility of the studies, and discrepancies were resolved by consensus.

## 3 Azvudine for treating COVID-19

### 3.1 Pharmacokinetics

**FIGURE 2:**
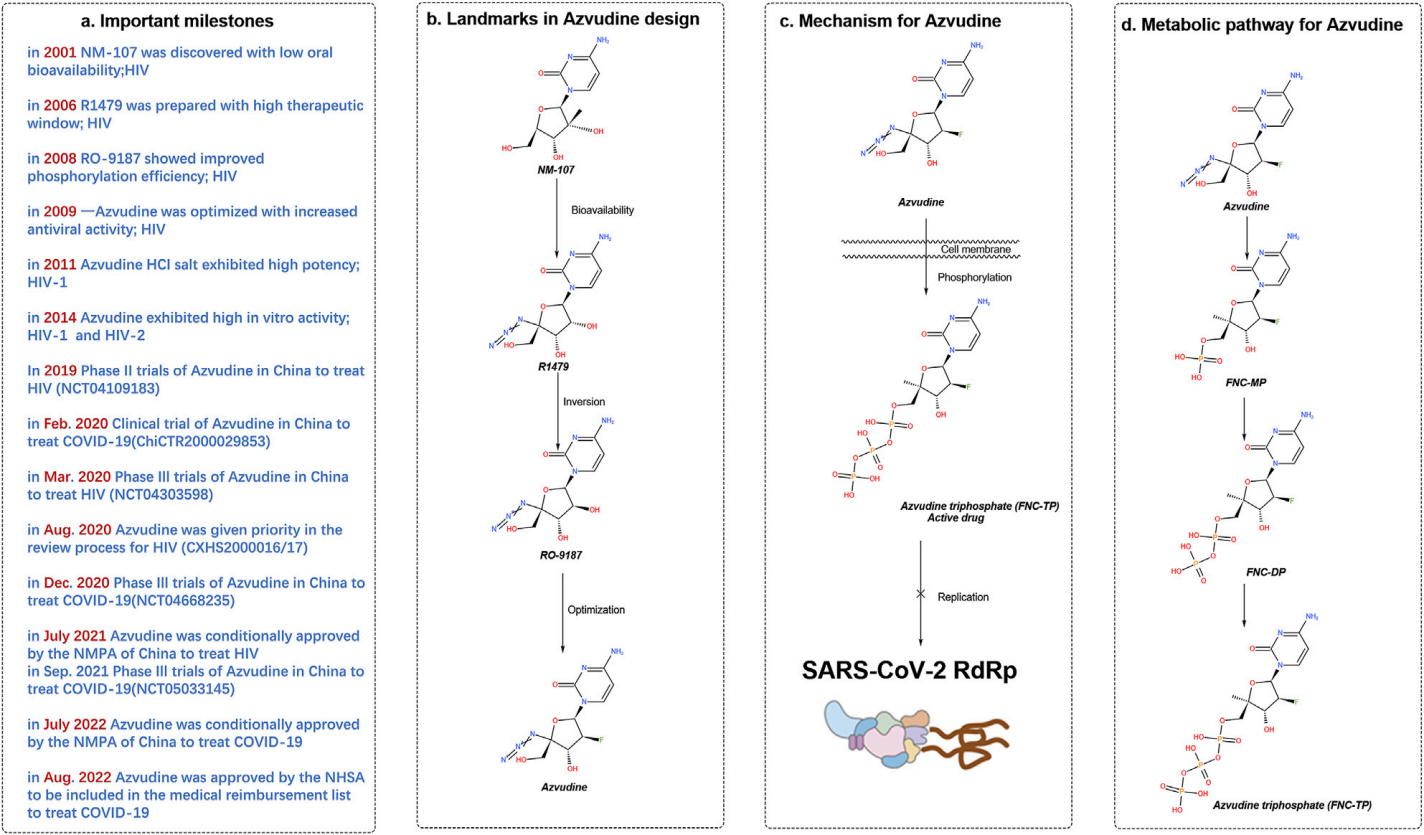
History of the discovery of azulfidine, an oral SARS-CoV-2 RdRp covalent inhibitor. **(A)** Important milestones in the discovery of azivudine from NM-107 to the first-in-class dual HIV and SARS-CoV-2 inhibitor. **(B)** Advances in medicinal chemistry led to the discovery of the oral RdRp inhibitor azivudine. **(C)** Proposed mechanism of action of the prodrug azivudine against SARS-CoV-2. **(D)** Metabolic pathway of azulfidine. The SARS-CoV-2 RNA replication process is blocked by the active metabolite, azulfidine triphosphate, through the formation of covalent bonds. Created with BioRender software (https://app.biorender.com/)

The synthesis of azvudine began with structural modification and optimization of the anti-HIV RdRp inhibitor RO-9187, which is derived from the potent nucleoside inhibitor NM-107 (Sommadossi and La Colla, 2001a; Sommadossi and La Colla, 2001b) (Figure 2). The 4′-azido-substituted R1479 exhibited significant activity against HIV *in vitro* (IC50 = 1.28 μM), with increased oral bioavailability and a larger therapeutic window (Klumpp et al., 2006; Smith et al., 2007). The 2′-hydroxyl inversion to form RO-9187 resulted in increased anti-HIV potency *in vitro* (IC50 = 0.171 μM) (Klumpp et al., 2008; Smith et al., 2009a). Additionally, RO-9187 showed increased phosphorylation efficiency, a rate-limiting step in the process. Replacing the 2′-β-hydroxyl group of RO-9187 with 2′-β-fluorine increased the *in vitro* anti-HCV potency (EC50 = 0.024 μM) (Smith et al., 2009b). The formation of FNC hydrochloride resulted in excellent antiviral activity against HIV (wild-type) *in vitro* (EC50 = 0.13 nM) (Wang et al., 2011). FNC also demonstrated excellent *in vitro* activity against HIV-1 (EC50 = 0.03–6.92 nM) and HIV-2 (EC50 = 0.018–0.025 nM) with low cytotoxicity (selectivity index [SI] > 1000) (Wang et al., 2014a). The safety and effectiveness of FNC have been evaluated in HIV patients since 2013, with several clinical trials (e.g., ClinicalTrials.gov: NCT04109183, NCT04303598) demonstrating its favorable long-term safety profile in a 48-week oral regimen for patients with AIDS (Zhang et al., 2021b). FNC was conditionally approved by the State Drug Administration of China on 21 July 2021 (XZXK-2021-214), for treating HIV (Yang and Wang, 2023).

FNC exhibits broad-spectrum antiviral properties *in vitro*, including activity against HIV-1 (EC50 = 0.03–6.92 nM), HIV-2 (EC50 = 0.018–0.025 nM), HCoV-OC43 (EC50 = 4.3 μM), and SARS-CoV-2 (EC50 = 1.2 μM). It has shown good anti-SARS activity in infected rhesus monkeys with favorable anti-SARS-CoV-2 activity. Notably, FNC targeted the thymus in a rat SARS-CoV-2 model and showed excellent absolute oral bioavailability (F = 82.7%) in a dog model.

In a clinical trial involving HIV-infected patients, continuous oral administration of azvudine for 7 days in two groups (2 mg twice daily and 4 mg once daily) resulted in some accumulation of azvudine in the body in the 2 mg group but not in the 4 mg group. Adverse events (AEs) were similar between fasting and postprandial administration (Ren et al., 2020a). Compared with fasting administration, postprandial administration significantly increased the exposure of the body to azvudine. Hence, it is recommended that azvudine be taken on an empty stomach. After a single oral dose in rats, azvudine showed the greatest distribution in the thymus (Zhang et al., 2021c), followed by the spleen, with lower levels in the heart, liver, and lungs. Azvudine had low plasma protein binding in humans, dogs, and rats.

The pharmacokinetic data of azvudine in COVID-19 treatment revealed excellent pharmacokinetics in different phases of clinical trials (GQ-FNC-2014-2, GQ-FNC-201, and NCT04109183), and other available information was derived from animal studies and HIV-infected patient trials.

Azvudine is primarily excreted unchanged through the kidneys. In HIV-infected patients, more than 70% of the drug is excreted within 12 h postadministration. Increased doses resulted in increased urinary excretion.

### 3.2 Pharmacodynamics

Azvudine inhibits SARS-CoV-2 and HCoV-OC43 coronaviruses (Chen et al., 2020) with an EC50 of 1.2–4.3 μmol/L, depending on the virus or cell type and a selection index (SI) of 15–83. The active triphosphate form of azvudine accumulates mainly in the thymus and peripheral blood mononuclear cells (PBMCs) in rats. In rhesus monkeys infected with SARS-CoV-2, azvudine reduced the viral load, restored thymic immunity, improved lymphocyte distribution, and reduced inflammation and organ damage (Zhang J. L. et al., 2021).

### 3.3 Clinical effectiveness evaluation

The primary pathophysiological mechanism of COVID-19 involves viral replication in the early days of infection and the subsequent host immune-inflammatory response (Ledford and Maxmen, 2022). Early administration of antiviral drugs or neutralizing antibodies and anti-inflammatory drugs in moderate to severe cases is crucial.

Clinical studies have shown that azvudine shortens the time to nucleic acid negativity compared with standard antiviral treatment (Zhou et al., 2023). In a randomized, open, controlled clinical trial of azvudine for mild and moderate COVID-19, the average time to consecutive negative nucleic acid tests was reduced by 4.5 days compared with that of standard treatment, with no observed drug-related adverse reactions (Ren et al., 2020a). Azvudine also showed significant clinical benefits in specific subgroups, such as males, those under 65 years of age, patients hospitalized for over 5 days, patients with severe COVID-19, and those receiving antibiotic treatment (Zhang J. L. et al., 2021).

The results of studies conducted in various countries revealed the effectiveness of azvudine in reducing the viral load and improving clinical symptoms. In Russia, the proportion of subjects with clinical improvement was significantly greater in the azvudine group than in the control group on the 7th day after administration (azvudine group: 57/157; control group: 15/157; P < 0.001). The median time to clinical improvement was also significantly shorter (azvudine group: 10 days; control group: 13 days; P < 0.001) (Hkexnews, 2024). In China, one study revealed no statistically significant difference in viral load changes, but another reported greater RT‒PCR negativity in the azvudine group, and the median RT‒PCR negativity time was significantly shorter (azvudine group: 2.6 days; control group: 5.6 days; P = 0.008). In Brazil, studies have indicated shorter times to first RT‒PCR negativity (azvudine group: 6.24 days, control group: 7.94 days, P = 0.002) and shorter hospital stays (azvudine group: 6.5 days, control group: 7.73 days, P = 0.028) for azvudine-treated patients (Ren et al., 2020a; da Silva et al., 2023) (Table 2).

**TABLE 2:** Characteristics of the published studies of clinical trials of azvudine.

Article	Clinical trials registration	Phase	Center	Study population	Study design		Results	
					Intervention group	Control group	Primary outcome	Secondary outcome
(de Souza et al., 2023)	NCT04668235	3	Single-center	Patients with moderate COVID-19	Azvudine 5 mg + standard treatment (n = 89)	Placebo + standard treatment (n = 83)	Shorter time of the first RT-PCR negativity in the Azvudine group than the control group (Azvudine group: 6.24 days, control group: 7.94 days, *P* = 0.002)	Shorter time of hospital stay (Azvudine group: 6.5 days, control group: 7.73 days, *P* = 0.028)
(da Silva et al., 2023)	NCT05033145	3	Single-center	Patients with mild COVID-19	Azvudine 5 mg + standard treatment (n = 143)	Placebo + standard treatment (n = 138)	Higher proportion of RT-PCR negativity in the azvudine group than the control group	Shorter median time of RT-PCR negativity (Azvudine group: 2.6 days, control group: 5.6 days, *P* = 0.008)
(Ren et al., 2020b)	ChiCTR2000029853	3	Single-center	Patients with mild and common COVID-19	Azvudine 5 mg + standard treatment (n = 10)	Placebo + standard treatment (n = 10)	Shorter mean times of the first nucleic acid negative conversion in the azvudine group than the control group (2.60 days vs 5.60 days, *P* = 0.008)	Higher nucleic acid negativity conversion rate in the azvudine group than in the control group
Russia, 2022 (a)	NR	NR	Multi-center	Patients with moderate COVID-19	Azvudine 5 mg (n = 157)	Placebo (n = 157)	Higher clinical improvement rate on the 7th day after the first administration of azvudine (Azvudine group: 57/157, control group: 15/157, *P* < 0.001)	Shorter median time of clinical improvement (Azvudine group: 10 days, control group: 13 days, *P* < 0.001)

A recent study evaluating the clinical effectiveness of azvudine in hospitalized COVID-19 patients revealed benefits in males under 65 years of age, those hospitalized for more than 5 days post-symptom onset, patients with severe COVID-19 at admission, and those receiving antibiotic treatment at admission. The study included 228 azvudine recipients and 228 nirmatrelvir/ritonavir recipients, revealing that the incidence rates of composite disease progression were lower in the azvudine group (6.662 per 1,000 person-days) than in the nirmatrelvir/ritonavir group (13.493 per 1,000 person-days, *P* = 0.029). There were no statistically significant differences between the groups in all-cause mortality (*P* = 0.183), noninvasive respiratory support (*P* = 0.068), the intubation rate (*P* = 0.144), or the ICU admission rate (*P* = 0.144). Cumulative risk analysis revealed a lower risk of composite disease progression in the azvudine group than in the nirmatrelvir/ritonavir group (HR: 0.51, 95% CI: 0.28–0.95; *P* = 0.029). These findings suggest the potential of azvudine as an effective treatment in specific subgroups of hospitalized COVID-19 patients, warranting further investigation and potential clinical practice integration (Dian et al., 2023; Gao et al., 2023).

In another retrospective cohort study, azvudine demonstrated significant clinical effectiveness in reducing disease progression risk, although the difference in all-cause mortality was not statistically significant (Sun et al., 2023a; Zong et al., 2023; Sun et al., 2023b). A real-world retrospective cohort study in China revealed greater clinical benefits in hospitalized patients with COVID-19 aged <65 years, those with a history of disease, those with severe COVID-19 at admission, and those receiving antibiotics of azvudine than nirmatrelvir-ritonavir (Deng et al., 2023). Some main retrospective studies on the clinical effectiveness of azvudine versus paxlovid are shown in Table 3. A meta-analysis revealed that azvudine and paxlovid had similar effectiveness in reducing mortality rates, negative PCR conversion times and hospital stays. However, azvudine was more effective at improving other outcomes (Amani and Amani, 2024).

**TABLE 3:** Clinical effectiveness of Azvudine versus Paxlovid including main retrospective studies.

First author	Year	Country	Data collection period	Sample size	Male %	Severity of COVID-19	Azvudine				Paxlovid				Results
							*N*	Mean age	Comorbidity (%)	COVID-19 vaccination	*N*	Mean age	Comorbidity (%)	COVID-19 vaccination	
Deng et al. (2023)	2023	China	December 2022 to January 2023	562	60	mild to severe	281	67.5	83.3	49.1	281	67.4	84.3	49.1	The lower crude incidence rate of composite disease progression outcome (7.83 vs 14.83 per 1000 person-days, p = 0.026) and all-cause death (2.05 vs 5.78 per 1000 person-days, p = 0.052) were observed among Azvudine recipients. Azvudine was associated with lower risks of composite disease progression outcome (hazard ratio [HR]: 0.55; 95% confidence interval [CI]: 0.32–0.94) and all-cause death (HR: 0.40; 95% CI: 0.16–1.04)
Dian et al. (2023)	2023	China	December 2022 to January 2023	456	64	mild to severe	228	69.21	100	48.2	228	70.44	100	48.2	No statistical differences between these two groups in the rates of all-cause death (P = 0.183), non-invasive respiratory support (P = 0.068), initiation of endotracheal intubation (P = 0.144), intensive care unit admission (P = 0.989) (Figure 2A). Cumulative hazard analysis demonstrated that patients treated with Azvudine had lower risk of composite disease progression outcome than those treated with Paxlovid (hazard ratio [HR]: 0.51; 95% CI: 0.28–0.95, P = 0.029)
Fu et al. (2023)	2023	China	December 2022 and January 2023	140	64	mild to critical	62	45.0	69.4	NR	49	50	59.2	NR	no significant differences in other clinical data, ICU admission or death between the three groups
Gao et al. (2023)	2023	China	NR	134	60	Not reported	67	70.5	NR	NR	67	70.2	NR	NR	Patients who received nirmatrelvir-ritonavir tended to have a shorter period to the first RT-PCR negative conversion than those who received azvudine (days, 5.8 [95% CI, 4.7–7.4] vs 10.0 [95% CI, 8.2–11.7])
Zhao et al. (2023a)	2023	China	December 2022 and January 2023	286	61	mild to severe	143	76.4	34.2	NR	143	76.8	35.6	NR	No significant differences in all-cause mortality (HR 1.41; 95% CI 0.56-3.56; P = 0.471), risk of progressing to critical COVID-19 (HR 1.67; 95% CI 0.78-3.60; P = 0.189), proportion with nucleic-acid negative conversion (HR 0.87; 95% CI 0.69-1.09; P = 0.220), length of stay (β - 0.82; 95% CI - 2.78 to 1.15; P = 0.414) and adverse event rate (3.21% vs 4.41%, P = 0.538) between the two groups
Shao et al. (2023)	2023	China	December 2022 to February 2023	966	61	severe to critical	177	78	NR	34.7	280	78	NR	34.7	No significant difference between azvudine and paxlovid in terms of time to sustained clinical recovery (p = 0.429) and death rates (p = 0.687)
Wei et al. (2023)	2023	China	December 2022 and January 2023	725	64	mild to critical	461	68	24	NR	264	65	25	NR	No significant difference in the composite disease progression events between azvudine (98, 21.26%) and nirmatrelvir/ritonavir (72, 27.27%) groups (p = 0.066). Azvudine was associated with a significant reduction in secondary outcomes, including the percentage of intensive care unit admission (p = 0.038) and the need for invasive mechanical ventilation (p = 0.035), while the in-hospital death event did not significantly differ (p = 0.991)
Zhao et al. (2023b)	2023	China	August 2022 to September 2022	227	46	mild to critical	82	51	28.1	78	145	46.5	19.3	81.4	No significant difference between the mean nucleic acid test negative conversion time was comparable between the nirmatrelvir-ritonavir and azvudine groups (7.0 [11.0, 15.0] vs 9.0 [6.0, 12.0] days, P = 0.064)
Zhou et al. (2023)	2023	China	December 2022 to January 2023	1154	61	mild to severe	311	70	35.7	NR	165	73	35.7	NR	Azvudine reduced all-cause death (Hazard ratio [HR]: 0.31; 95% CI: 0.12–0.78), and its composite with invasive mechanical ventilation (HR: 0.47; 95% CI: 0.24–0.92)

The inconsistent conclusions regarding the effectiveness of Azvudine and Paxlovid in Table 3 are primarily due to multiple factors. In terms of study design, differences exist in sample size, data collection periods, and types of studies. Regarding statistical methods, variations in analytical models and definitions of endpoint events are observed. Some studies conducted simple comparative analyses, while others employed complex multivariate regression models. Additionally, the definitions of endpoint events, such as “disease progression,” vary in strictness across studies. According to the studies in Table 3, Azvudine has demonstrated effectiveness in hospitalized patients with severe conditions, older age, and comorbidities, reducing mortality and the risk of disease progression. In particular, its effectiveness is comparable to that of Paxlovid in patients with severe COVID-19.

Regarding the inconsistency of the previous studies, in 2025 a new meta-analysis that included 21 studies covering 10,011 patients confirmed that compared with standard care and Paxlovid (nirmatrelvir/ritonavir) control groups, Azvudine significantly reduced the mortality risk of COVID-19 patients (Wang et al., 2025). The mortality rate in the Azvudine group was significantly lower than that in the standard care/placebo group (RR = 0.48, 95% CI: 0.40–0.57, P < 0.001). The mortality rate in the Azvudine group was also significantly lower than that in the Paxlovid group (RR = 0.73, 95% CI: 0.58–0.92, P < 0.05). In summary, Azvudine has shown significant promise as a treatment for COVID-19, particularly in high-risk patients, by reducing mortality, decreasing viral load, and improving immune response.

Azvudine was approved for import registration for treating common COVID-19 patients with high-risk factors. On 25 July 2022, it was formally launched as the second small-molecule oral medication for COVID-19 in mainland China. On 6 January 2023, the People’s Republic of China’s National Health Commission included azvudine in the Scheme for Diagnosis and Treatment of SARS-CoV-2 (The 10th Trial Edition). Compared with the 9th Edition of the previously released diagnosis and treatment plan, in addition to paxlovid (Hammond et al., 2022), monoclonal antibodies, intravenous injections of human immunoglobulin and convalescent plasma, and antiviral treatment include the addition of azvudine.

Expanding on these findings, previous studies have primarily focused on the original SARS-CoV-2 strain. A new study of characteristics of patients with non-severe infections of different SARS-CoV-2 omicron subvariants in China with 244 Omicron (BA.2.76 and BA.5.1) infected patients showed more frequent clinical symptoms and higher viral loads, but shorter viral clearance times. Azulfidine is safe and effective in the treatment of SARS-CoV-2 omicron subvariants, shortening the viral clearance time, increasing the levels of antiviral antibodies and immune cells, and decreasing the levels of inflammatory factors (Yuan et al., 2024). This latest study fills a research gap by demonstrating that Azvudine is also effective against Omicron variants.

### 3.4 Safety

Azvudine’s technical evaluation report for marketing approval mentioned its genotoxicity and reproductive toxicity (Center for drug evaluation, 2024). In Ames tests, CHL chromosome aberration tests, and *in vivo* mouse micronucleus tests, azvudine had positive results. It affects the ovarian mass and increases fetal resorption rates in rats but does not significantly harm male fertility. Clinical application has reported mild adverse reactions such as fever, headache, dizziness, nausea, vomiting, and diarrhea (Zhang J. L. et al., 2021). Severe adverse reactions are rare, and the drug has a good safety profile, with no significant impact on underlying diseases or elderly patients; however, liver and kidney functions should be closely monitored during treatment.

A meta-analysis including 5 RCTs (Ren et al., 2020a; Hkexnews, 2024; da Silva et al., 2023) reported that the incidence of adverse events in COVID-19 patients in the intervention groups who received azvudine treatment was 44.52% (256/575) and that in the control groups was 49.74% (282/567). The incidence of serious adverse events in COVID-19 patients in the intervention groups who received azvudine treatment was 1.16% (5/432), and that in the control groups was 1.86% (8/429). The safety of azvudine was better than that in the control group (adverse events: RR = 0.89, 95% CI: 0.80--0.99, P = 0.04; serious adverse events: RR = 0.63, 95% CI: 0.22--1.79, P = 0.39) (Chen and Tian, 2023; Zheng et al., 2024).

Further supporting Azvudine’s favorable safety profile, another mata-analysis comparing Azvudine with Paxlovid also showed Azvudine has better safety profile overall. The meta-analysis including 21 studies of 10,011 patients collected data on adverse events (AEs) during follow-up for both the azvudine and Paxlovid groups (Wang et al., 2025). Compared with azvudine recipients, patients in the Paxlovid group had a greater risk of Grade 1 AEs, including increased alanine aminotransferase (ALT) (*p* = 0.013), hypercholesterolemia (*p* < 0.001), and increased aspartate aminotransferase (AST) (*p* = 0.047). With respect to Grade 2 AEs, Paxlovid administration was related to greater risks of increased decreased platelet (*p* = 0.009), increased creatinine (*p* = 0.018), and ALT (*p* = 0.036) than azvudine. For Grade 3 and greater SEs, Paxlovid treatment was related to a higher incidence of decreased lymphocyte count (*p* < 0.001).

Recent cases have indicated that azvudine could cause sinus tachycardia (Ren et al., 2020a). It did not cause renal-related adverse events in COVID-19 patients but requires more information on its use in patients with a GFR ≤60 mL/min/1.73 m2.

### 3.5 Drug interactions

Azvudine is a substrate and weak inducer of P-gp (Liu et al., 2017; Liu et al., 2018). Caution is needed when P-gp substrates, inhibitors, and inducers are coadministered. Azvudine modulates the expression of P-gp, MRP2, and BCRP, affecting their absorption and potentially enhancing the antiviral activity of other antiretroviral drugs. Azvudine has demonstrated synergistic therapeutic effects on the EC50 against HIV-1IIIB-infected C8166 cells and HIV-1TC-1-infected PBMCs when used in combination with six FDA-approved antiretroviral drugs (Wang et al., 2014b).

Azvudine’s drug interaction profile is an important consideration for its clinical use. When combined with other COVID-19 drugs, such as immunosuppressants or anticoagulants, there may be potential risks. The use of azvudine with anticoagulants may require careful monitoring due to the potential for increased bleeding risk. Further research is needed to fully understand these interactions and to provide guidance on the safe use of azvudine in combination with other medications.

### 3.6 Special populations

#### 3.6.1 Pregnant or lactating women, children, and elderly patients

Currently, there are no studies on the use of azvudine in pregnant or lactating women, and it is not recommended for these populations. Effective contraception is advised for women of childbearing age during treatment and for 4 days after the last dose.

Clinical studies in children and elderly individuals are lacking. It is generally not recommended for use in individuals under 18 years old. Elderly patients, especially those with underlying conditions, should be closely monitored for liver and kidney function during treatment.

#### 3.6.2 High-risk factor patients

For the treatment of common COVID-19 patients with advanced severe high-risk factors, such as advanced age, not having received the COVID-19 vaccine, chronic kidney disease, diabetes, severe cardiovascular disease, chronic obstructive pulmonary disease, organ transplant recipients, and other individuals taking immunosuppressive medications (Ju et al., 2024), azvudine has been approved for import registration by the Scheme for Diagnosis and Treatment of SARS-CoV-2 (The 10th Trial Edition) published on 6 January 2023, by the People’s Republic of China’s National Health Commission.

For those high risk patients such as elderly patients (>60 years) and those with primary malignant tumors, the subgroup analysis from a meta-analysis suggesting a greater benefit of azvudine over Paxlovid. Subgroup analysis of a meta-analysis including 21 studies of 10,011 patients showed in elder patients (>60 years) (Wang et al., 2025), for all-cause death, potentially meaningful interactions suggesting a greater benefit of azvudine over Paxlovid in those with primary malignant tumors (p for interaction <0.001, HR: 0.33, 95% CI: 0.20–0.54), and in those without systemic steroid use (p for interaction = 0.004, HR: 0.67, 95% CI: 0.53–0.84). For composite disease progression, potentially meaningful interactions suggesting a greater benefit of azvudine over Paxlovid were observed in patients with moderate COVID-19 (p for interaction = 0.036, HR: 0.67, 95% CI: 0.45–1.00) and with primary malignant tumors (p for interaction = 0.012, HR: 0.54, 95% CI: 0.33–0.88). No differences of all-cause death between the azvudine and paxlovid group were observed in severe covid-19, diabetes, hypertension, liver diseases, cardio-cerebral diseases, kidney diseases, autoimmune diseases, and chronic respiratory diseases patients.

The recommended doses of azvudine for COVID-19 patients at different stages of chronic kidney disease (CKD) are listed in Table 4 (Kale et al., 2023).

**TABLE 4:** Recommended dose of azvudine in COVID-19 patients with different stages of CKD.

Drug name	CKD stages	Recommended dose	Adverse effects
Azvudine	G1	100%	Dizziness and nausea in the approx. 10% of patients
	G2	100%	
	G3	100%	
	G4	Lower dose is recommended	
	G5	Can be given with caution	
	hemodialysis	Can be given with caution	
	Risk for nephrotoxicity	Not reported	
	Risk for accumulation and systemic toxicity	Not reported	

#### 3.6.3 Hemodialysis patients

For hemodialysis patients with COVID‐19, a Chinese multicenter observational study revealed that the negative nucleic acid conversion rate of the azvudine group was significantly greater than that of the basic treatment group. There were no significant differences in liver function, renal function, or the number of adverse events between the two groups, suggesting that azvudine may be safe and effective compared with the basic treatment of hemodialysis patients with common COVID‐19 (Shang et al., 2023).

#### 3.6.4 Other special patients

Patients with a history of pancreatitis or viral hepatitis should use azvudine cautiously because of its structural similarities with lamivudine, a nucleoside reverse transcriptase inhibitor known to cause pancreatitis in some cases. Severe and potentially fatal liver events have been reported in patients with chronic hepatitis B or C coinfection with HIV who are receiving antiretroviral therapy (Thompson et al., 2015; Köklü et al., 2013; Park et al., 2011).

## 4 Antiviral and antitumor activities of azvudine

**FIGURE 3:**
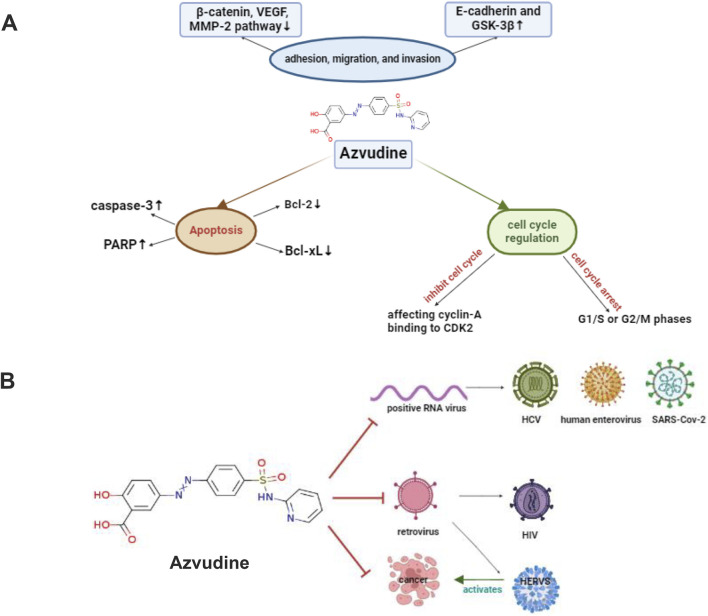
Overview of how FNC fights tumors through various pathways **(A)** FNC induces cell death by lowering Bcl-xL and Bcl-2 levels and activating caspase-3, which breaks down key proteins like PARP. FNC also prevents Raji and JeKo-1 cells from sticking, moving, and invading by increasing E-cadherin and GSK-3β and decreasing β-catenin, VEGF, MMP-2, and MMP-9. The Wnt/β-catenin pathway is important in cancer growth, and changes in this pathway are linked to tumor invasiveness. FNC causes cell cycle arrest at G1/S or G2/M phases and induces apoptosis. It stops cell cycle checkpoints, like Cyclin-A binding to CDK2, allowing cells to finish the S phase and move to the M phase **(B)** Graphic Abstract, shows the mechanisms of FNC anticancer and antiviral activities. Abbreviations*HIV: human immunodeficiency virus, HCV: hepatitis C virus, HERVs: human endogenous retroviruses. Created with BioRender software (https://app.biorender.com/)

Azvudine has broad-spectrum antiviral activity against RNA viruses, such as human immunodeficiency virus (HIV), hepatitis C virus (HCV), enterovirus 71 (EV71), and hepatitis B virus (HBV) (Zhang J. L. et al., 2021). It also has underlying antitumor mechanisms through various pathways. Figure 3 shows how FNC might work against cancer and viruses.

It shows potent inhibitory activity against both wild-type and drug-resistant HIV strains, with EC50 values ranging from 0.063 nM to 0.735 nM. In 2021, the National Medical Products Administration (NMPA) approved its use to treat HIV-1 infected adult patients [63].

Azulfidine also has antitumor potential, which can modulate the tumor immune microenvironment and inhibit tumor growth. For example, azulfidine significantly inhibited the proliferation and invasive ability of hepatocellular carcinoma (HCC) cells in in vitro experiments (Wang et al., 2025). A subgroup analysis of a Meta-study showed that azulfidine demonstrated greater benefit in the treatment of patients with malignant tumors, significantly reducing the composite of all-cause mortality (HR = 0.33, 95% CI: 0.20–0.54) and disease progression (HR = 0.54, 95% CI: 0.33–0.88). *In vitro* experiments showed that azulfidine significantly inhibited the proliferation and invasive ability of HCC cell lines and lung cancer cell lines, whereas Paxlovid did not. In in vivo experiments, azulfidine significantly inhibited H22 cell-induced tumor growth in mice with a favorable safety profile. Single-cell RNA sequencing showed that the proportion of CD4^+^ T cells and CD8^+^ T cells in the tumor immune microenvironment increased after azulfidine treatment, and specific subpopulations of these cells exhibited functional alterations, suggesting that azulfidine may exert its antitumor effects by modulating immune cells. These findings suggest that azulfidine not only has a promising application in antiviral therapy, but may also provide a new strategy for tumor therapy.

## 5 Conclusion

Azvudine shows promise as an effective treatment for COVID-19, particularly in reducing disease progression and viral load, with a favorable safety profile characterized by mild and transient side effects. However, current research is limited by small sample sizes, which may affect the generalizability of results, and a lack of long-term effectiveness and safety data, restricting its widespread clinical application. Future studies should focus on evaluating Azvudine’s effectiveness against different SARS-CoV-2 variants, exploring its potential for combination therapies, and developing second-generation inhibitors. Additionally, conducting larger clinical trials targeting high-risk populations, such as the elderly, immunocompromised individuals, and those with severe renal impairment, will provide stronger evidence to support its clinical use and optimize treatment protocols.
